# Effects of exercise training on markers of adipose tissue remodeling in patients with coronary artery disease and type 2 diabetes mellitus: sub study of the randomized controlled EXCADI trial

**DOI:** 10.1186/s13098-019-0508-9

**Published:** 2019-12-19

**Authors:** Hani Zaidi, Rune Byrkjeland, Ida U. Njerve, Sissel Åkra, Svein Solheim, Harald Arnesen, Ingebjørg Seljeflot, Trine B. Opstad

**Affiliations:** 1 Center for Clinical Heart Research, Department of Cardiology, Oslo University Hospital Ullevål, Nydalen, PB 4956, 0424 Oslo, Norway; 2 Center for Heart Failure Research, Oslo University Hospital, Oslo, Norway; 3 Faculty of Medicine, University of Oslo, Oslo, Norway

**Keywords:** MMP-9, TIMP-1, EMMPRIN, Galectin-3, Exercise, Remodeling, Adipose-tissue

## Abstract

**Background:**

Investigate effects of long-term exercise on the remodeling markers MMP-9, TIMP-1, EMMPRIN and Galectin-3 in combined type 2 diabetes mellitus (T2DM) and coronary artery disease (CAD) patients. Any associations between these biomarkers and glucometabolic variables were further assessed at baseline.

**Methods:**

137 patients (age 41–81 years, 17.2% females) were included and randomized to a 12-months exercise program or to a control group. Fasting blood samples and subcutaneous adipose tissue (AT) samples were taken at inclusion and after 12-months. The intervention was a combination of aerobic and strength training for a minimum of 150 min per week. Circulating protein levels were measured by ELISA methods and RNA was extracted from AT and circulating leukocytes. Expression levels were relatively quantified by PCR.

**Results:**

After 12 months of intervention, both AT-expression and circulating levels of EMMPRIN were increased in the exercise group (*p* < 0.05, both) with significant difference in change between the two groups (*p* < 0.05 both). No significant effect was observed on MMP-9, TIMP-1 and Galectin-3. Levels of TIMP-1 (AT-expression and circulating) were significantly correlated to insulin, and HOMA2- after Bonferroni correction (p = 0.001, by 48 performed correlations).

**Conclusion:**

The increase in levels of EMMPRIN after long-term exercise training, might indicate some degree of AT remodeling in these patients after 12-months of exercise, whether beneficial or not. The remodeling markers were to some extent associated with glucometabolic variables in our population with the combined disease.

*Trial registration* clinicaltrials.gov, NCT01232608. Registered 2 November 2010

## Background

Adipose tissue (AT) is a dynamic and a potentially pro-inflammatory organ, especially in obese individuals [1]. Depending on the stimuli it receives, the AT may undergo extensive remodeling, with extracellular matrix (ECM) degradation and infiltration of macrophages and other pro-inflammatory cells, creating both a local and a systemic inflammatory environment with subsequent production of pro-inflammatory mediators [2]. This may precipitate decreased insulin-sensitivity and subsequently type 2 diabetes mellitus (T2DM) and coronary artery disease (CAD) [3]. To permit changes in shape, size and number of adipocytes, the ECM has to reorganize. Though timely degradation of ECM is a necessity for tissue repair and physiological development, a dysregulated, uncontrolled degradation of the ECM may lead to pathological conditions such as fibrosis, atherosclerosis and heart failure [4].

Matrix metalloproteinase (MMP)-9, which is produced by many different cell types including neutrophils, macrophages and fibroblasts [5], is one of the main components in ECM degradation and thereby believed to promote AT remodeling [1] and plaque rupture [6]. MMP-9 seems to be particularly related to cardiovascular disease (CVD) risk factors and metabolic syndrome (MetS) [7]. Furthermore, there seems to be an association between body mass index (BMI) and plasma levels of MMP-9, suggesting AT as a potential source [8].

Tissue inhibitor of matrix metalloproteinase-1 (TIMP-1) regulates MMP-9 activity, by forming MMP9–TIMP1 complexes [9]. TIMP-1 is independently related to cardiovascular events and to some glucometabolic variables because of its broad range of action apart from MMP-9 inhibition [10], including cardiac remodeling [11]. MMP-9 and TIMP-1 are both induced by the protein extracellular matrix metalloproteinase inducer (EMMPRIN) [12]. Reports on EMMPRIN suggest that it is involved in the development of CVD and myocardial infarction (MI), in part through promoting expression of pro-inflammatory chemokines and platelet activation [13]. EMMPRIN is also involved in tissue remodeling, not only through induction of MMPs, but also through mechanisms such as promotion of myofibroblast differentiation [14]. EMMPRIN has been shown to be expressed in atherosclerotic plaques as well as in different cell types such as endothelial cells, epithelial cells and leukocytes [15].

Galectin-3 is considered to be a marker of fibrosis and remodeling [16] and associated with obesity, inflammation, T2DM and circulating levels of Galectin-3 [17, 18]. It is produced in many different cell-types, including macrophages and adipocytes, [17] and inhibition of Galectin-3 has been shown to prevent AT remodeling in obese rats [19].

The beneficial effects of exercise is evident, through lowering the risk of CVD, by reducing several cardiovascular risk factors such as obesity, hypertension, T2DM and MetS [20]. Furthermore, exercise has been shown to have beneficial effects on AT remodeling in rats [21]. The effect of exercise training in humans on MMP-9 and TIMP-1 is not entirely clear. Studies suggest that the response on MMP-9 is dependent on the type and duration of the exercise, indicating that an acute bout of exercise might cause an increase in plasma levels of MMP-9, whereas long term resistance-exercise may lead to a decrease, with no clear effect on TIMP-1 [22–24]. Limited data exist on the effects of long-term exercise on EMMPRIN and Galectin-3.

We therefore aimed to explore the effects of exercise training on MMP-9, TIMP-1, EMMPRIN and Galectin-3 expression in AT in patients with combined CAD and T2DM, and whether their expression in AT corresponded to circulating levels of the proteins as well as expression levels in circulating leukocytes. Any associations to glucometabolic variables at baseline were further explored.

We hypothesized that exercise-training would modify the gene- and protein expression of the investigated markers of fibrosis and tissue remodeling.

## Methods

This is a sub-study of the EXCADI (Exercise training in patients with coronary artery disease and type 2 diabetes) trial, in which patients with T2DM and CAD (n = 137) were included at the department of Cardiology, Oslo University Hospital, Ullevaal, Norway, between August 2010 and March 2012. The study design has previously been described [25]. In brief, 137 subjects were randomized 1:1 to an exercise group or a control group. The exercise group was assigned to a 12-month exercise program, consisting of 150 min of strength and endurance exercise per week, and the control group was followed up in a conventional manner by their General Practitioner for 12-months. All subjects had known T2DM and stable CAD verified by coronary angiography. Hypertension was defined as those on antihypertensive medication and smoking history was defined as those that were either current or previous smokers. Advanced vascular disease was defined as those who previously had suffered a MI and/or diabetic microvascular complications (defined as a history of nephropathy, neuropathy or retinopathy, and/or abnormal monofilament test and/or (micro-) albuminuria). In the initial study, increased anaerobic threshold and time to exhaustion were observed in the exercise group, and when stratified for patients with advanced vascular disease, a significant increase in VO_2max_ and a decrease in HbA1c were observed in patients without advanced vascular disease, as previously published [25]. We have therefore used the same definition for patients with advanced vascular disease in the present study.

Exclusion criteria were presence of proliferative retinopathy, end-stage renal disease, cancer, stroke or acute MI within the last 3 months, unstable angina, decompensated heart failure, serious arrhythmia, severe valvular disease, severe rheumatologic disease, chronic obstructive pulmonary disease stadium GOLD IV, thromboembolic disease, ongoing infections, severe musculoskeletal disorders and other disabilities limiting the ability for physical activity.

The Regional Ethics Committee approved the study and all patients gave their written informed consent to participate. The study was conducted in accordance to the Declaration of Helsinki.

### Physical exercise intervention

The physical exercise program was developed in collaboration with the Norwegian School of Sport Sciences. The details of the program have been described previously [25]. Briefly, the exercise group had two group based sessions with strength and endurance exercise and one home-based session, amounting to a total of 150 min of exercise per week. Approximately two-thirds of the exercise was aerobic and one-third was resistance training.

### Laboratory methods

Venous blood samples were collected by standard venipuncture between 08.00 a.m. and 10.00 a.m. in fasting condition, before intake of morning medication both at inclusion and after 12 months. AT was taken from the gluteal region and snap frozen immediately at − 80 °C until RNA was extracted. PAXgene tubes (PreAnalytix GmbH, Hombrechtikon, Switzerland) were collected for RNA extraction from circulating leukocytes. Blood samples, including HbA1c, insulin and C-peptide, were acquired and determined through conventional methods. Serum was prepared by centrifugation within 1 h at 2500×*g* in 10 min for determination of circulating MMP-9, TIMP-1, EMMPRIN and Galectin-3, measured by the following ELISA kits: Human MMP-9, Human TIMP-1, Human EMMPRIN/CD147 and Human Galectin 3, all R&D Systems Europe (Abingdon, Oxon UK). The inter-assay CVs in our laboratory were 8.3%, 5.6%, 4.3% and 5.6%, respectively.

Total RNA was extracted using the PAXgene^®^ Blood RNA Kit (produced by Qiagen GmbH, PreAnalytix, Hilden, Germany), with an extra cleaning step (RNeasy^®^ MinElute^®^ Cleanup Kit; Qiagen). RNA quality and quantity (ng/µL) were determined by the NanoDrop™ 1000 Spectrophotometer (Nanodrop technologies, DE, USA). Total RNA from AT was isolated, including disruption and homogenization in Tissue lyser (Qiagen), by use of a high Pure RNA Tissue Kit (Hoffman-La Roche Ltd., Basel, Switzerland), according to a combination of the kit protocol and previous experience in our laboratory. RNA extracted from both sources were stored at − 80 °C until analyses. Copy DNA (cDNA) was synthesized from equal amount of RNA with qScript™ cDNA superMix (Quanta Biosciences Inc., Gaithersburg, USA). Real-time PCR was performed on a ViiA™7 instrument, using TaqMan^®^ Universal PCR Master Mix (P/N 4324018) and TaqMan^®^ assays for MMP-9 (Hs00234579_m1), TIMP-1 (Hs00171558_m1), EMMPRIN (Hs00936295_m1) and Galectin-3 (Hs00173587_m1) (Applied Biosystems, by Life Technologies, Foster City, CA, USA). β-2-microglobulin (Hs99999907_m1) (Applied Biosystems) was used as the endogenous control, and mRNA levels were determined by relative quantification (RQ) using the ΔΔCT method [26].

### Statistical analyses

Demographic data are given as proportions, or median (25th and 75th percentiles) for skewed data. Differences between groups for continuous variables were analyzed by Mann–Whitney U test, and Chi square test was used for categorical variables. Within-group changes after intervention were analyzed by Wilcoxon Signed Rank test, and differences in change between the randomized groups were performed by Mann–Whitney U test. Baseline associations were analyzed by Spearmans’s rho correlations, and multiple comparisons were adjusted for by Bonferroni correction. Statistical calculations were performed using SPSS version 25 (SPSS Inc., Chicago, Illinois, USA). p-values < 0.05 were defined as statistically significant.

## Results

Baseline characteristics of the total population and according to the randomized groups are shown in Table 1.

**Table 1:** Baseline characteristics of the total study population (n = 137) and according to the randomized groups

	All (137)	Exercise (52)	Control (62)
Age	64 (58, 69)	65 (58, 70)	64 (60, 68)
Sex (m/f)	115/22	45/7	51/11
Previous AMI, n (%)	62 (45)	20 (39)	31 (49)
Advanced vascular disease, n (%)^a^	79 (57)	28 (54)	41 (66)
CHF, n (%)	11 (8)	2 (4)	5 (8)
PAD, n (%)	13 (9, 5)	3 (6)	6 (10)
Years with DM	9.0 (5, 15)	11.0 (5.0, 15.0)	9.0 (5.5, 13.5)
Hypertension, n (%)	100 (73)	39 (75)	48 (76)
Current smokers, n (%)	23 (16.8)	9 (17)	9 (14)
SBP (mmHg)	138 (127, 150)	136 (129, 150)	140 (126, 150)
DBP (mmHg)	79 (71, 86)	76 (71, 82)	81 (71, 87)
Weight (kg)	86.5 (77.1, 97.0)	87.0 (78.5, 99.0)	85.0 (77.5, 96.0)
HbA1c (%)	7.4 (6.8, 8.3)	7.4 (6.8, 8.4)	7.4 (6.8, 8.0)
Insulin (pmol/L)	57 (33, 101)	54 (31, 95)	63 (32, 104)
C-peptide (pmol/L)	965 (713, 1290)	956 (637, 1165)	1042 (743, 1453)
Total cholesterol (mmol/L)	3.9 (3.4, 4.6)	3.8 (3.4, 4.3)	4.0 (3.3, 4.8)
Triglycerides (mmol/L)	1.42 (1.06, 1.91)	1.44 (1.09, 1.86)	1.36 (0.99, 1.88)
LDL (mmol/L)	2.0 (1.6, 2.6)	1.8 (1.5, 2.5)	2.2 (1.6, 2.9)
HOMA2-IR	1.3 (0.7, 2.1)	1.1 (0.7, 1.9)	1.3 (0.7, 2.2)
BMI (kg/m^2^)	28.7 (25.7, 31.6)	29.4 (25.5, 31.8)	28.1 (25.6, 31.6)
Medication, n (%)			
ACE-inhibitors	43 (31.6)	14 (27)	21 (34)
A2-blockers	55 (40.1)	20 (38)	25 (40)
Statins	128 (93.4)	49 (94)	59 (95)
Metformin	101 (73.7)	40 (77)	46 (74)
Sulfonylureas	48 (35.0)	23 (44)	18 (29)
Gliptin	17 (12.4)	6 (12)	11 (18)
Insulin	26 (19.1)	12 (17.4)	14 (20.9)
Anti-platelet drugs	129 (94)	47 (90)	61 (98)*

Values are given as number (proportions), or median (25, 75 percentile)

*AMI* acute myocardial infarction, *SBP* systolic blood pressure, *DBP* diastolic blood pressure, *DM* diabetes mellitus, *LDL* low-density lipoprotein, *CHF* congestive heart failure, *PAD* peripheral artery disease, *HOMA2-IR* homeostatic model assessment indexes-insulin resistance, *BMI* body mass index, *ACE* angiotensin converting enzyme, *A2* angiotensin II

p-values refer to differences between the exercise and control group

* p = 0.054

^a^Advanced vascular disease is defined as those with previous MI and/or diabetic microvascular complications in addition to CAD

Of the 137 included patients, 123 completed the study and 9 patients with the lowest adherence to the intervention principle were excluded [25]. Thus, a total of 114 patients were analyzed for the intervention effect. No significant differences in baseline characteristics between the randomized groups were observed, except for borderline significant larger proportion of anti-platelet users in the control group.

Blood samples were available in all at baseline, whereas after intervention, the numbers of successfully analyzed samples were 114 and 107 for circulating MMP-9, TIMP-1, EMMPRIN and Galectin-3 and their gene expression in leukocytes, respectively. Numbers of successfully analyzed samples in AT ranged from 55 to 100. The limited number of AT samples was mainly because of patients’ unwillingness to give fat tissue biopsies, particularly after the intervention period, and inadequate quantity and quality of the samples.

As previously reported, there were no significant between-group differences in changes in weight, waist circumference, energy intake percentages of main nutrients or diabetes medication during the study period [25].

### Effects of exercise training

Levels of the remodeling markers in the exercise and control group before and after the intervention period are shown in Table 2.

**Table 2:** Levels of remodeling markers at baseline and after 12-months of intervention in the randomized groups

	Control			Exercise			∆p	Relative ∆p
	Baseline	12-months	p^1^	Baseline	12-months	p^2^		
AT-MMP9	1.98 (0.89, 3.26)	1.61 (0.97, 3.50)	0.586	1.57 (0.82, 3.05)	1.18 (0.89, 1.91)	0.184	0.650	0.767
AT-TIMP1	1.02 (0.79, 1.15)	1.08 (0.79, 1.43)	0.476	0.96 (0.75, 1.30)	0.99 (0.80, 1.45)	0.495	0.908	0.929
AT-EMMPPRIN	0.99 (0.73, 1.28)	0.82 (0.61, 1.25)	*0.021*	0.86 (0.73, 1.06)	0.95 (0.66, 1.26)	0.146	0.008*	0.018*
AT-Galectin-3	1.03 (0.69, 1.38)	0.96 (0.64, 1.30)	*0.015*	1.05 (0.83, 1.31)	1.00 (0.70, 1.24)	0.179	0.366	0.366
L-MMP9	0.79 (0.57, 1.30)	0.72 (0.47, 1.47)	0.837	0.86 (0.60, 1.58)	0.77 (0.61, 1.27)	0.158	0.480	0.544
L-TIMP1	0.98 (0.68, 1.61)	1.04 (0.67, 1.64)	0.654	1.09 (0.88, 1.71)	0.96 (0.70, 1.63)	0.313	0.822	0.434
L-EMMPRIN	0.66 (0.36, 1.00)	0.61 (0.27, 1.24)	0.599	0.63 (0.45, 1.03)	0.60 (0.35, 0.82)	*0.035*	0.271	0.388
L-Galectin-3	0.94 (0.68, 1.35)	0.94 (0.55, 1.41)	0.273	1.04 (0.71, 1.35)	0.80 (0.61, 1.29)	*0.030*	0.284	0.411
sMMP9	317 (198, 416)	288 (189, 431)	0.956	308 (215, 427)	335 (257, 490)	0.054	0.152	0.052
sTIMP1	158 (140, 175)	160 (143, 184)	0.328	158 (140, 184)	168 (149, 191)	*0.001*	0.066	0.082
sEMMPRIN	3748 (3432, 4118)	3707 (3515, 4100)	0.908	3848 (3391, 4404)	3983 (3452, 4521)	*0.007*	0.033*	0.036*
sGalectin-3	10.6 (8.1, 12.7)	10.0 (8.3, 11.6)	0.806	10.3 (8.3, 11.7)	10.2 (8.6, 12.3)	0.074	0.138	0.246

Values are median (25, 75 percentiles)

*AT* refers to gene expression in adipose tissue, *L* refers to gene expression in circulating leukocytes, *s* indicate serum

p^1^-values refer to changes within the control group from baseline to 12 months (Wilcoxon Signed Rank test)

p^2^-values refer to changes within the exercise group from baseline to 12 months (Wilcoxon Signed Rank test)

p-values in italics indicate statistically significant values

Delta p (Δp) refers to difference in change between the groups during intervention (Mann–Whitney test)

Relative delta p (Δp) refers to difference in change between the groups during intervention as related to baseline levels

#### Gene expression in AT

Expression of EMMPRIN increased significantly in the exercise group compared to the control group after 12 months (Δ*p* = 0.008*, relative* Δ*p* = 0.018). Galectin-3 expression decreased significantly in the control group, however, the change when compared to the exercise group, did not reach statistical significance (Δ*p* = 0.366). MMP-9 and TIMP-1 did not change significantly during the intervention.

#### Gene expression in circulating leukocytes

We observed a significant decrease in the expression of EMMPRIN and Galectin-3 after 12-months in the intervention group, however, not significantly different from the controls. No significant difference in changes in MMP-9 and TIMP-1 were observed.

#### Circulating levels of the markers

Circulating levels of EMMPRIN increased significantly in the exercise group compared to the control group after 12 months (*∆p* = 0.033, *relative* Δ*p* = 0.036). There was also a significant increase in the circulating levels of TIMP-1 (*p* = 0.001) and a borderline significant increase in MMP-9 (*p* = 0.054) in the intervention group, however, the difference in change between the intervention group and the control group did not reach statistical significance (Δ*p* = 0.066 and Δ*p* = 0.152, respectively). No intervention effects were observed for Galectin-3.

#### Patients with advanced vascular disease

When analyzing separately the subgroup of patients with advanced vascular disease (n = 79) according to the intervention effect, the increase in circulating levels of TIMP-1 was statistically significant in the intervention group compared to the control group (Δ*p* = 0.002), and a more pronounced difference in change, with significantly more increased levels of circulating EMMPRIN in the intervention group (Δ*p* < 0.001) was noted. No further significant differences between the groups were observed (data not shown).

### Baseline correlations to glucometabolic variables (Table 3)

We found some associations between the glucometabolic variables insulin, C-peptide, HOMA2-IR and BMI and the investigated markers EMMPRIN and TIMP-1. However, after a Bonferroni correction (p = 0.001, by 48 performed correlations) only the correlation between circulating levels of TIMP-1 to insulin and HOMA2-IR, remained statistically significant (p ≤ 0.001).

**Table 3:** Correlations between remodeling markers and glucometabolic variables

	Diabetes duration	Glucose	HbA1c	Insulin	C-peptide	HOMA2-IR	BMI
AT-MMP-9	r = 0.007 p = 0.951	r = 0.173 p = 0.130	r = 0.073 p = 0.522	r = 0.099 p = 0.383	r = 0.052 p = 0.647	r = 0.110 p = 0.348	r = 0.057 p = 0.618
AT-TIMP-1	r = − 0.152 p = 0.142	r = 0.069 p = 0.508	r = 0.092 p = 0.368	r = 0.290 p = 0.004	r = 0.302 p = 0.003	r = 0.226 p = 0.032	r = 0.236 p = 0.020
AT-EMMPRIN	r = − 0.041 p = 0.695	r = 0.062 p = 0.550	r = 0.019 p = 0.855	r = − 0.063 p = 0.544	r = − 0.239 p = 0.019	r = − 0.045 p = 0.674	r = − 0.048 p = 0.639
AT-Galectin-3	r = − 0.055 p = 0.571	r = − 0.036 p = 0.715	r = − 0.072 p = 0.457	r = 0.008 p = 0.933	r = 0.036 p = 0.713	r = − 0.011 p = 0.914	r = − 0.070 p = 0.473
s-MMP-9	r = − 0.090 p = 0.304	r = 0.096 p = 0.266	r = 0.245 p = 0.004	r = 0.039 p = 0.652	r = − 0.109 p = 0.205	r = 0.071 p = 0.427	r = − 0.004 p = 0.967
s-TIMP-1	r = 0.074 p = 0.397	r = 0.120 p = 0.165	r = 0.054 p = 0.529	*r* *=* *0.274* *p* *=* *0.001*	r = 0.175 p = 0.041	*r* *=* *0.321* *p* *<* *0.001*	r = 0.278 p = 0.002
s-EMMPRIN	r = 0.170 p = 0.049	r = − 0.064 p = 0.461	r = − 0.023 p = 0.792	r = 0.232 p = 0.006	r = 0.213 p = 0.012	r = 0.264 p = 0.003	r = 0.206 p = 0.022
s-Galectin-3	r = 0.114 p = 0.189	r = 0.008 p = 0.924	r = 0.039 p = 0.653	r = 0.095 p = 0.267	r = 0.118 p = 0.169	r = 0.118 p = 0.188	r = − 0.011 p = 0.941

*AT* gene-expression in adipose tissue, *L* gene-expression in leukocytes, *s*; serum

Italics p-values indicate positive correlation after Bonferroni correction (p = 0.001)

### Inter-correlations between the investigated remodeling markers (Additional file 1: Table S1)

Expression of the remodeling markers in AT were neither correlated to their circulating levels nor to their respective gene expression in circulating leukocytes. When looking at the correlations between the markers within the same compartments, levels of MMP-9 and TIMP-1 were inter-correlated in all 3 compartments, levels of TIMP-1 and EMMRIPN were inter-correlated in the circulation and in circulating leukocytes, whereas expression of Galectin-3 was inter-correlated with expression of TIMP-1 and EMMPRIN in AT.

## Discussion

The main findings in our study were that long-term exercise intervention increased circulating levels and AT expression of EMMPRIN. Circulating TIMP-1 and MMP-9 (borderline significant) increased after the exercise period, but the difference in change between groups did not reach statistical significance. Circulating levels and AT expression of EMMPRIN and TIMP-1 correlated to glucometabolic variables, and AT expression of the markers were not reflected in their circulating levels or leukocyte expression.

Based on our results, the role of MMP-9 as a potential remodeling marker in AT is not apparent. Exercise training, in particular aerobic interval training, has been reported to reverse harmful remodeling of AT in high fat diet induced obesity in rats, by observed decreased systemic insulin resistance, decreased adipocyte size and increased capillary density in the AT [21]. It seems like chronic aerobic training, to a higher degree, also induces a reduction in circulating MMP-9 levels [23]. Whether this effect reflects exercise induced remodeling that happens in the AT is not fully understood. It is also inevitable to question if the amount of exercise in our study was sufficient to see any effects on the MMP-9 levels. The patients’ weight did not change significantly after 12 months of intervention [25], it is therefore unclear whether the AT went through a remodeling process that would be reflected in modified levels of MMP-9.

Circulating EMMPRIN increased significantly in the exercise compared to the control group. Because EMMPRIN is primarily an inducer of MMP-9, elevated levels of MMP-9 was expected, and MMP-9 did increase borderline significantly after exercise, however, not differently from the control group. Some evidence indicate that EMMPRIN also induces TIMP-1 [27], and accordingly, a simultaneous and significant increase in circulating TIMP-1 levels in the exercise group was observed, eventually inducing beneficial MMP-9 inhibition, however, the difference in change compared with the control group, was only borderline significant. The significantly increased AT expression of EMMPRIN in the exercise group compared to the control group, suggests that the exercise protocol in our study did affect the AT to some extent, but it is unclear whether it is in a beneficial direction or not. Overexpression of EMMPRIN has been observed in many inflammatory diseases, such as rheumatoid arthritis and systemic lupus erythematosus, after vascular injury and in atherosclerotic plaques [15]. An association between EMMPRIN expression in leukocytes and CAD has been reported, and lastly, left ventricular remodeling has been shown to be associated with myocardial EMMPRIN expression [28, 29]. This is indicative of EMMPRIN being a marker of adverse remodeling, particularly in the myocardium, and a marker of a pro-inflammatory state with increased risk of developing CAD [13]. One could therefore speculate whether 150 min of strength and endurance exercise per week is healthy or maybe even hazardous in this patient group with long-standing diabetes and CAD. This was further reflected in patients with advanced vascular disease, in which the increase of circulating levels of EMMPRIN was more pronounced in the exercise group after 12-months.

No significant effect of exercise intervention was observed on Galectin-3 in either compartment. Higher plasma levels of Galectin-3 have been shown in patients with unstable angina and were also correlated with the number of diseased coronary vessels [30]. Previous studies have reported increased plasma levels of Galectin-3 immediately after strenuous exercise, but no change in plasma levels was seen after long-term exercise (8 weeks) in healthy individuals [31]. This is consistent with our results, though our study population suffers from extensive comorbidity and was exposed for an extended exercise program. Considering that Galectin-3 is presumably a marker of fibrosis, it is not unlikely that persisted elevated levels in our population may reflect an irreversible change, which could explain the lack of exercise effects on this marker.

Continuous elevated levels of glucose may lead to glycation of proteins and disrupt their normal functions by altering enzymatic activity and interfere with receptor functioning [32]. We therefore wanted to explore any correlations between the measured remodeling markers and glucometabolic variables, however, only limited associations were observed. This may be due to the heavy medical regimens in all patients for both their diseases. The significant correlation between circulating levels of TIMP-1 to insulin, and HOMA2-IR, might nevertheless, reflect that remodeling in AT is related to insulin resistance and MetS [33]. When investigating inter-correlations between the remodeling markers, we observed that their gene expression in AT were not reflected in the corresponding circulating levels or leukocyte expression, maybe indicating other potentially more important sources. The consistent inter-correlation between all markers in circulating leukocytes and between MMP-9 and TIMP1 in all compartments may confirm inter-regulatory relationships, partly cell-specific. Galectin-3′ correlation to TIMP-1 and EMMPRIN in AT expression may indicate potential common remodeling signaling pathways.

Most exercise studies performed on patients with advanced CAD or T2DM are in agreement about the beneficial effects of exercise training, mostly through lowering of modifiable CAD risk factors, improvement of glycemic control and increase in exercise capacity [34, 35]. However, these studies do not address the impact of exercise training on remodeling in the AT.

The importance of understanding the role of MMP-9 and its regulators has previously been emphasized, as it may potentially open up novel therapeutic approaches for treating CVD and metabolic diseases [13, 36]. Further studies, maybe in a healthier population, are warranted in order to shed more light on the effects of exercise training on AT.

### Limitations

This study was initially designed for the purpose of measuring effects on HbA1c after exercise training [25], hence, the initial power calculations have not considered effects on the investigated remodeling markers. The reluctance of some patients to give AT samples after 12-months, lead to a limited number of samples post-intervention. Our population consisted of heavily medically treated patients with long-standing disease, and about 60% had developed advanced vascular disease with vascular complications. One might question if the remodeling in AT had reached an irreversible state rendering the assigned physical activity not to be beneficial in these particular patients. Any physical activity in the control group was not accounted for, which may have implications on the results. The strength of this study was the randomization principle with equal distribution of patients and few differences between the randomized groups.

## Conclusion

The increase in AT expression and in circulating levels of EMMPRIN after long-term exercise in patients with CAD and long-standing T2DM with microvascular complications indicate that some degree of AT remodeling occurs in this patient population, whether beneficial or not. The limited association between the remodeling markers and glucometabolic variables might be because all patients were well treated for their disease.

## Supplementary information

**Additional file 1.** Intercorrelations between the investigated markers.

## Data Availability

The datasets used and analyzed during the current study are available from the corresponding author on reasonable request.

## Abbreviations

AT: adipose tissue
BMI: body mass index
CAD: coronary artery disease
cDNA: copy DNA
CVD: cardiovascular disease
ECM: extracellular matrix
EMMPRIN: extracellular matrix metalloproteinase inducer
HOMA2-IR: homeostatic model assessment 2 of insulin resistance
MMP: matrix metalloproteinase
MetS: metabolic syndrome
MI: myocardial infarction
T2DM: type 2 diabetes mellitus
TIMP-1: tissue inhibitor of matrix metalloproteinase

## References

[1] Kumari M, Heeren J, Scheja L (2018). Regulation of immunometabolism in adipose tissue. Semin Immunopathol.

[2] Itoh M, Suganami T, Hachiya R (2011). Adipose tissue remodeling as homeostatic inflammation. Int J Inflam.

[3] Tanaka M, Itoh M, Ogawa Y (2018). Molecular mechanism of obesity-induced ‘metabolic’ tissue remodeling. J Diabetes Investig.

[4] Visse R, Nagase H, Murphy G (2006). Structure and function of matrix metalloproteinases and TIMPs. Cardiovasc Res.

[5] Yabluchanskiy A, Ma Y, Iyer RP (2013). Matrix metalloproteinase-9: many shades of function in cardiovascular disease. Physiology (Bethesda, Md).

[6] Loftus IM, Naylor AR, Goodall S (2000). Increased matrix metalloproteinase-9 activity in unstable carotid plaques. A potential role in acute plaque disruption. Stroke.

[7] Yu AP, Tam BT, Yau WY (2015). Association of endothelin-1 and matrix metallopeptidase-9 with metabolic syndrome in middle-aged and older adults. Diabetol Metab Syndr.

[8] Laimer M, Kaser S, Kranebitter M (2005). Effect of pronounced weight loss on the nontraditional cardiovascular risk marker matrix metalloproteinase-9 in middle-aged morbidly obese women. Int J Obes.

[9] Ries C (2014). Cytokine functions of TIMP-1. Cell Mol Life Sci.

[10] Lindsey ML, Yabluchanskiy A, Ma Y (2015). Tissue inhibitor of metalloproteinase-1: actions beyond matrix metalloproteinase inhibition. Cardiology.

[11] Opstad TB, Seljeflot I, Bohmer E (2018). MMP-9 and its regulators TIMP-1 and EMMPRIN in patients with acute ST-elevation myocardial infarction: a NORDISTEMI substudy. Cardiology.

[12] Mishra B, Kizaki K, Sato T (2012). The role of extracellular matrix metalloproteinase inducer (EMMPRIN) in the regulation of bovine endometrial cell functions. Biol Reprod.

[13] von Ungern-Sternberg SNI, Zernecke A, Seizer P (2018). Extracellular matrix metalloproteinase inducer EMMPRIN (CD147) in cardiovascular disease. Int J Mol Sci.

[14] Huet E, Gabison EE, Mourah S (2008). Role of emmprin/CD147 in tissue remodeling. Connect Tissue Res.

[15] Wang C, Jin R, Zhu X (2015). Function of CD147 in atherosclerosis and atherothrombosis. J Cardiovasc Translation Res.

[16] Li LC, Li J, Gao J (2014). Functions of galectin-3 and its role in fibrotic diseases. J Pharmacol Exp Ther.

[17] Weigert J, Neumeier M, Wanninger J (2010). Serum galectin-3 is elevated in obesity and negatively correlates with glycosylated hemoglobin in type 2 diabetes. J Clin Endocrinol Metab.

[18] Rhodes DH, Pini M, Castellanos KJ (2013). Adipose tissue-specific modulation of galectin expression in lean and obese mice: evidence for regulatory function. Obesity (Silver Spring, Md).

[19] Martinez-Martinez E, Calvier L, Rossignol P (2005). Galectin-3 inhibition prevents adipose tissue remodelling in obesity. Int J Obes.

[20] Agarwal SK (2012). Cardiovascular benefits of exercise. Int J Gen Med.

[21] Kolahdouzi S, Talebi-Garakani E, Hamidian G (2019). Exercise training prevents high-fat diet-induced adipose tissue remodeling by promoting capillary density and macrophage polarization. Life Sci.

[22] Jaoude J, Koh Y (2016). Matrix metalloproteinases in exercise and obesity. Vasc Health Risk Manag.

[23] Nascimento Dda C, Durigan Rde C, Tibana RA (2015). The response of matrix metalloproteinase-9 and -2 to exercise. Sports Med.

[24] Lo Presti R, Hopps E, Caimi G (2017). Gelatinases and physical exercise: a systematic review of evidence from human studies. Medicine.

[25] Byrkjeland R, Njerve IU, Anderssen S (2015). Effects of exercise training on HbA1c and VO_2_ peak in patients with type 2 diabetes and coronary artery disease: a randomised clinical trial. Diabetes Vasc Dis Res.

[26] Livak KJ, Schmittgen TD (2001). Analysis of relative gene expression data using real-time quantitative PCR and the 2(-Delta Delta C(T)) Method. Methods.

[27] Reddy VS, Prabhu SD, Mummidi S (2010). Interleukin-18 induces EMMPRIN expression in primary cardiomyocytes via JNK/Sp1 signaling and MMP-9 in part via EMMPRIN and through AP-1 and NF-kappaB activation. Am J Physiol Heart Circ Physiol.

[28] Spinale FG, Coker ML, Heung LJ (2000). A matrix metalloproteinase induction/activation system exists in the human left ventricular myocardium and is upregulated in heart failure. Circulation.

[29] Pennings GJ, Yong AS, Kritharides L (2010). Expression of EMMPRIN (CD147) on circulating platelets in vivo. J Thromb Haemo.

[30] Falcone C, Lucibello S, Mazzucchelli I (2011). Galectin-3 plasma levels and coronary artery disease: a new possible biomarker of acute coronary syndrome. Int J Immunopathol Pharmacol.

[31] Moghadasi M (2017). Effect of 8 weeks regular endurance training on galectin-3 changes after a strenuous aerobic exercise. J Phys Act Hormones.

[32] Singh VP, Bali A, Singh N (2014). Advanced glycation end products and diabetic complications. Korean J Physiol Pharmacol.

[33] Matulewicz N, Stefanowicz M, Nikolajuk A (2017). Markers of adipogenesis, but not inflammation, in adipose tissue are independently related to insulin sensitivity. J Clin Endocrinol Metab.

[34] Chudyk A, Petrella RJ (2011). Effects of exercise on cardiovascular risk factors in type 2 diabetes: a meta-analysis. Diabetes Care.

[35] Gielen S, Laughlin MH, O’Conner C (2015). Exercise training in patients with heart disease: review of beneficial effects and clinical recommendations. Prog Cardiovasc Dis.

[36] Johnson JL (2017). Metalloproteinases in atherosclerosis. Eur J Pharmacol.

