# Supplementary material for: Effects of exercise training on markers of adipose tissue remodeling in patients with coronary artery disease and type 2 diabetes mellitus: sub study of the randomized controlled EXCADI trial
Source: Diabetol Metab Syndr. 2019 Dec 19;11:109. doi: 10.1186/s13098-019-0508-9 (PMC6923919; doi:10.1186/s13098-019-0508-9)
Supplement: Supplementary file 1 — Additional file 1. Intercorrelations between the investigated markers. [file 13098_2019_508_MOESM1_ESM.docx]

|  | AT MMP9 | AT TIMP-1 | AT EMMPRIN | AT Gal-3 | PAX MMP-9 | PAX TIMP-1 | PAX EMMPRIN | PAX Gal-3 | sMMP9 | sTIMP1 | sEMMPRIN | sGal-3 |
| --- | --- | --- | --- | --- | --- | --- | --- | --- | --- | --- | --- | --- |
| AT MMP9 | r=1.000 |  |  |  |  |  |  |  |  |  |  |  |
| AT TIMP-1 | r=0.408** | r=1.000 |  |  |  |  |  |  |  |  |  |  |
| AT EMMPRIN | r=0.019 | r=0.156 | r=1.000 |  |  |  |  |  |  |  |  |  |
| AT Gal-3 | r=0.216 | r=0.440** | r=0.557** | r=1.000 |  |  |  |  |  |  |  |  |
| PAX MMP-9 | r=--0.140 | r=-0.165 | r=0.015 | r=-0.099 | r=1.000 |  |  |  |  |  |  |  |
| PAX TIMP-1 | r=-0.083 | r=-0.079 | r=0.113 | r=0.015 | r=0.587** | r=1.000 |  |  |  |  |  |  |
| PAX EMMPRIN | r=0.117 | r=0.026 | r=0.049 | r=-0.042 | r=0.398** | r=0.580** | r=1.000 |  |  |  |  |  |
| PAX Gal-3 | r=0.031 | r=0.131 | r=0.029 | r=0.133 | r=0.198* | r=0.413** | r=0.736** | r=1.000 |  |  |  |  |
| sMMP9 | r=-0.108 | r=0.077 | r=-0.111 | r=-0.136 | r=0.158 | r=0.030 | r=-0.092 | r=-0.133 | r=1.000 |  |  |  |
| sTIMP-1 | r=-0.019 | r=0.007 | r=-0.123 | r=-0.188 | r=-0.166 | r=-0.073 | r=-0.163 | r=-0.197* | r=0.230** | r=1.000 |  |  |
| sEMMPRIN | r=0.068 | r=0.053 | r=-0.146 | r=-0.063 | r=0.030 | r=0.000 | r=0.042 | r=-0.027 | r=-0.023 | r=0.446** | r=1.000 |  |
| sGal-3 | r=-0.043 | r=0.008 | r=-0.095 | r=0.137 | r=-0.012 | r=-0.031 | r=-0.041 | r=0.122 | r=0.053 | r=0.146 | r=0.122 | r=1.000 |
|  |  |  |  |  |  |  |  |  |  |  |  |  |

Additional file 1 Intercorrelations between the investigated markers

AT; refers to gene expression in adipose tissue

PAX; refers to gene expression in leukocytes

s; indicate serum

* p<0.05

** p<0.01
